# Editorial: Privacy enhancing technology: a top 10 emerging technology to revolutionize healthcare

**DOI:** 10.3389/fdgth.2026.1782663

**Published:** 2026-03-25

**Authors:** Lisette J. E. van Gemert-Pijnen, Thijs Veugen

**Affiliations:** 1 University of Twente, Enschede, Netherlands; 2 University of Twente, Faculty Behavioural Management and Social Sciences, Enschede, Netherlands; 3 Unit ICT, Strategy and Policy, TNO, The Hague, Netherlands; 4 Department of Semantics, Cybersecurity and Services, University of Twente, Enschede, Netherlands

**Keywords:** differential privacy, federated learning, healthcare, privacy enhancing technologies (PETs), research agenda, synthetic data

## Background research topic privacy enhancing technologies (PETs)

PETs are one of the Top Ten Emerging Technologies of 2024, as announced in the report published by the World Economic Forum in collaboration with Frontiers (World Economic Forum, 2024)^1^.

The research topic explores the potentials of PETs to foster data utilization across institutions and nations, to promote precision medicine and personalized healthcare. Their objective is to safeguard privacy and security while enabling large-scale data utilization, processing, and analysis between different parties. By facilitating the utilization of vast datasets across institutions and nations, PETs are set to revolutionize multiple sectors, including healthcare, mobility, and energy. We refer to (1) for a classification and definition of PETs in light of this editorial.

## Perspective of PETs

To protect sensitive private data in healthcare, the step towards statistical tools is rather small as it closely aligns with known expertise and software used in the domain and they offer a fair level of data protection.

The six papers in our research topic nicely illustrate this approach and show how the different PETs relate to important developments in healthcare. This editorial highlights the synergy between the six papers and future challenges to apply PETs in the context of healthcare.

### Combination of different data sets

If multiple medical institutions, each having their own data source, want to combine their data for e.g., survival analysis, a federated and privacy-preserving variant of machine learning avoids centralizing all data. In Westers et al. they implement and evaluate a distributive protocol for Cox regression based on federated learning which shows that collaboration increases accuracy.

### Synthesizing data sets

For protecting the privacy of a single medical data source, and overcoming data scarcity, a way to go is synthesizing this data, i.e., artificially generating data that mimic the statistical properties of real data without revealing sensitive information. The use of synthetic data is further investigated for the purpose of rare disease research (Mendes et al.). The potential of the technique becomes clear and supports ensuring compliance with regulations like GDPR and HIPAA.

This is confirmed by a literature review (Jiang et al.) for the use of medical imaging, although there are some technical limitations for synthetically generating likely images. They also examine the role of synthetic medical image data within the European Health Data Space (EHDS), a policy initiative aimed at enabling secure access to health data across the EU. They provide an overview of European regulatory instruments to apply PETs, related to the ongoing debates on how synthetic imaging data can support a privacy-preserving, data-driven healthcare ecosystem in Europe.

Statistical techniques for generating synthetic data resemble the ones used in differential privacy: a method to add artificial noise to data records for hiding identifiable attributes (Hernandez et al.). Assesses the use of differential privacy in tabular data and finds that it is hard to measure its added value. They consider five generative models for creating synthetic data, a key technology in healthcare where many attributes are sensitive. Synthetic data also facilitates the training of AI models, simulation of clinical trials and cross border cooperation (Mendes et al.).

### Privacy and quality

In Hammer and Strufe they suggest adapting Differential Privacy, such that the mechanism can account for temporal context and correlations, making it suitable for time-continuous data, such as electrocardiographic (ECG) records. Such an anonymization mechanism is necessary in the context of European Health Data Space.

Synthetic data aligns with regulations as EHDS but should avoid reidentification risks and conflicts with GDPR's protection of individual rights and freedom (Mendes et al.). Although synthetic data can replicate real world data, the question remains to what extent quality can be maintained because not all nuances for high research value may not be captured in the generated data set. This means that hybrid models and a mixed method approach combining real world data with synthetic data are relevant for reasons of validity and clinical values (Mendes et al.).

## Future of PETs

Challenges for using PETs are maintaining data quality, mitigation bias and overcoming computational demands (Mendes et al.), and alignment with regulations to avoid reidentification. Transparency of algorithms, data statistics, Third Party reviews and domain experts' evaluations (Mendes et al.) can ensure an adequate and valid reuse of the data sets.

A closer look at EHDS, and how PETs could and should support this, is presented in (van Drumpt et al.). They use semi-structured interviews to explore risks, challenges and gaps in the implementation of PETs within EHDS, leading to a research agenda that addresses important future research questions. PETs should be considered directly from start, following privacy by design principles, to ensure data protection is embedded into the development of tech-systems, PETs are no afterthought (van Drumpt et al.).

The use of PETs is hindered by technological and legal aspects, the so-called LEFT aspects (2), legal, ethical, financial and technology aspects of implementation of novel or emergent technologies in healthcare. In van Drumpt et al., the socio-technical and legal concerns are identified to apply PETs, and solutions are discussed from the perspective of governance and policy and technology. The technology aspects are meaningful to ensure data quality, to enable federated systems, homomorphic encryption, and to improve access and data minimalization. To address the critical issue of data accuracy in the EHDS, van Drumpt et al. proposes the integration of zero-knowledge proofs (ZKPs) for the verification of AI models. The societal aspects demonstrate the risk of reinforcing disparities rather than democratizing access to data, and the lack of clear definitions (data holder, informed consent etc.) that can lead to misinterpretations across EU-member states.

Investments in cloud-based platforms to support computing resources for reducing computational overhead and emerging technologies such as Generative Adversarial Networks (GANs) and Variational Autoencoders (VAEs) should be leveraged (Mendes et al.).

### Implementation of pets

Applying PETs demand for adequate business model to understand cost/benefits in healthcare practices and required resources and capacities to perform PETs (3). A multi stakeholder collaboration between PET-experts, policymakers, industry leaders, and healthcare professionals is needed to develop standards for applying PETs, regarding quality, governance, building trust (public, healthcare) and compliance with frameworks for better and smarter data reuse (based on all six papers).

### Research agenda

The future research agenda should explore legal, ethical financial and technology aspects that foster or hinder the implementation of PETs in Healthcare across boundaries (institutions, borders) to develop a multi-stakeholder approach for secure and safe data collaboration to develop adequate business models for sustainable implementation of PETs (4). Such a business model is essential to better understand the cost, benefits and ROI for stakeholders that are involved in the development and implementation of PETs (ref to scoping review).

## Footnotes

^1^ https://www.weforum.org/publications/top-10-emerging-technologies-2024/

## References

[1] Privacy-enhancing technologies (PETs). Information Commisioner's Office (19 June 2023). Available online at: https://ico.org.uk/for-organisations/uk-gdpr-guidance-and-resources/data-sharing/privacy-enhancing-technologies/ (Accessed March 12, 2026).

[2] Bente BE Beerlage-de Jong N Verdaasdonk RM van Gemert-Pijnen JEWC. Unveiling practical insights of eHealth implementation in Europe: a grey literature review on legal, ethical, financial, and technological (LEFT) considerations. Front Digit Health. (2025) 7:1575620. 10.3389/fdgth.2025.1575620 40896375 PMC12390996

[3] Schenker JL. *The Promise of Privacy Enhancing Technologies*. The Innovator (2025). Available online at: https://theinnovator.news/the-promise-of-privacy-enhancing-technologies/ (Accessed March 12, 2026).

[4] Bente BE Van Dongen A Verdaasdonk R van Gemert-Pijnen L. eHealth implementation in Europe: a scoping review on legal, ethical, financial, and technological aspects. Front Digit Health. (2024) 6:1332707. 10.3389/fdgth.2024.1332707 38524249 PMC10957613

